# Functional activity, functional connectivity and complex network biomarkers of progressive hyposmia Parkinson’s disease with no cognitive impairment: evidences from resting-state fMRI study

**DOI:** 10.3389/fnagi.2024.1455020

**Published:** 2024-09-25

**Authors:** Lei Geng, Wenfei Cao, Juan Zuo, Hongjie Yan, Jinxin Wan, Yi Sun, Nizhuan Wang

**Affiliations:** ^1^Department of Medical Imaging, The Second People’s Hospital of Lianyungang, Lianyungang, China; ^2^The Oncology Hospital of Lianyungang, Lianyungang, China; ^3^Lianyungang Clinical College of Jiangsu University, Lianyungang, China; ^4^Department of Neurology, Heze Municipal Hospital, Heze, China; ^5^Department of Ultrasound, The Fourth People’s Hospital of Lianyungang, Lianyungang, China; ^6^Department of Neurology, Affiliated Lianyungang Hospital of Xuzhou Medical University, Lianyungang, China; ^7^Department of Chinese and Bilingual Studies, The Hong Kong Polytechnic University, Kowloon, Hong Kong SAR, China

**Keywords:** Parkinson’s disease, hyposmia, amplitude of low-frequency fluctuation, regional homogeneity, functional connectivity, brain functional network

## Abstract

**Background:**

Olfactory dysfunction stands as one of the most prevalent non-motor symptoms in the initial stage of Parkinson’s disease (PD). Nevertheless, the intricate mechanisms underlying olfactory deficits in Parkinson’s disease still remain elusive.

**Methods:**

This study collected rs-fMRI data from 30 PD patients [15 with severe hyposmia (PD-SH) and 15 with no/mild hyposmia (PD-N/MH)] and 15 healthy controls (HC). To investigate functional segregation, the amplitude of low-frequency fluctuation (ALFF) and regional homogeneity (ReHo) were utilized. Functional connectivity (FC) analysis was performed to explore the functional integration across diverse brain regions. Additionally, the graph theory-based network analysis was employed to assess functional networks in PD patients. Furthermore, Pearson correlation analysis was conducted to delve deeper into the relationship between the severity of olfactory dysfunction and various functional metrics.

**Results:**

We discovered pronounced variations in ALFF, ReHo, FC, and topological brain network attributes across the three groups, with several of these disparities exhibiting a correlation with olfactory scores.

**Conclusion:**

Using fMRI, our study analyzed brain function in PD-SH, PD-N/MH, and HC groups, revealing impaired segregation and integration in PD-SH and PD-N/MH. We hypothesize that changes in temporal, frontal, occipital, and cerebellar activities, along with aberrant cerebellum-insula connectivity and node degree and betweenness disparities, may be linked to olfactory dysfunction in PD patients.

## 1 Introduction

Parkinson’s disease (PD), whose impact on society is significant, is a common neurodegenerative disease characterized by motor dysfunction, tremor, muscle rigidity, and non-motor symptoms (Reich and Savitt, 2019; Kalia and Lang, 2015). With population growth and aging, the epidemiology of Parkinson’s disease has changed considerably, and its incidence rate is increasing. A recent Global Burden of Disease (GBD) study showed that age-standardized incidence rates of Parkinson’s disease increased largely among nervous system diseases (Huang et al., 2023). However, the causes of Parkinson’s disease are complex and uncertain, and current researches suggest that genetics, environment, and interactions thereof may be involved (Bloem et al., 2021; Nonnekes et al., 2018; Nalls et al., 2019; Ryan et al., 2019; Skrahina et al., 2021). In clinical settings, Parkinson’s disease is primarily diagnosed through a comprehensive assessment of medical history and neurological symptoms (Armstrong and Okun, 2020). It’s worth noting that when clinical manifestations appear, at least 50–60% of dopaminergic neurons have already died, which can potentially delay treatment (Lang, 2007). The topic of motor symptoms, including tremor, rigidity, and slowness, is often crucial and cannot be overlooked. In addition to the motor features, there are also many non-motor features, such as olfactory dysfunction, cognitive decline, sleep disorders, depression and apathy (Grażyńska et al., 2020; Yan et al., 2023). Olfactory abnormalities usually appear before motor symptoms, known as “prodromal” symptoms, which means that changes in the olfactory pathway may be the earliest pathological changes in PD (Löhle et al., 2020).

As a subjective sensation, olfaction is inherently a chemical sensing process that involves the detection of specific odor molecules by sensory cells (Dan et al., 2021). The olfactory system comprises the peripheral olfactory system, such as the olfactory neuroepithelium and olfactory tract, as well as the central olfactory system, including the olfactory bulb and olfactory cortex (Hadley et al., 2004). The specific binding of odor ligands to receptors on olfactory neurons triggers the activation of the second messenger pathway, resulting in the generation of action potentials. Subsequently, these action potentials are transmitted in a graded fashion through the olfactory tract, ultimately reaching the olfactory bulb and olfactory cortex. This sequential process constitutes the fundamental mechanism underlying the production of olfactory sensation (Buck and Axel, 1991; Pinto, 2011). A fully functional olfactory system enables humans to assess the safety of consumed food and perceive vital information about surrounding dangers. However, this capability gradually diminishes as individuals age (Branigan and Tadi, 2023). Olfactory dysfunction, characterized by the diminution or absence of olfactory function, can be congenital, idiopathic, or associated with cognitive disorders (Fonteyn et al., 2014; Ramirez-Gomez et al., 2022; Wang et al., 2021; Shao et al., 2021). Among the prevalent causes of this condition are sinus diseases, infections, and head trauma (Huynh et al., 2020). Olfactory impairment stands as the most prevalent non-motor symptom among individuals with PD, affecting over 90% of patients who exhibit notable olfactory decrement (Nabizadeh et al., 2022). The impairment of odor recognition has been recognized as a pivotal central defect that characterizes both PD and Alzheimer’s disease (AD) (Dan et al., 2021). In addition, olfactory function testing can assist in the early diagnosis of PD, so a large number of researchers have attempted to focus on the study of olfactory dysfunction in PD patients (Fujio et al., 2020; Braak and Braak, 2000). However, the intricacies surrounding the mechanism of olfactory impairment in PD patients remain elusive, necessitating deeper investigation through rigorous molecular, pathological, and imaging research endeavors.

In recent years, deep learning-based methodologies and machine learning models have commenced being utilized to uncover biomarkers for the early detection of Parkinson’s disease (PD) (Guo et al., 2022; Chen et al., 2024; Tran et al., 2024; Mei et al., 2021). Despite the innovative nature of these methodologies, they are not without limitations. Specifically, they fall short in providing a holistic and multi-dimensional understanding of the intricate early brain alterations in PD patients. Magnetic Resonance Imaging (MRI) has been extensively employed to investigate the structural, functional, and metabolic changes in the brain linked to olfactory dysfunction in PD patients, thus facilitating the search for diagnostic biomarkers (Roh et al., 2021; Wang et al., 2011; Tanik et al., 2016; Roos et al., 2019; Su et al., 2015; Wang et al., 2022). Resting-state functional magnetic resonance imaging (rs-fMRI) is a widely utilized imaging technique for evaluating the dynamic alterations in brain function among patients in a resting state. Amplitude of low-frequency fluctuation (ALFF) (Zang et al., 2007), regional homogeneity (ReHo) (Zang et al., 2004), functional connectivity (FC) (van den Heuvel and Hulshoff Pol, 2010), and complex network analysis serves as integral components of the evaluation indicators (Bullmore and Sporns, 2009; Yan et al., 2022), enabling the exploration of alterations in brain function from both the angles of functional segregation and functional integration.

Therefore, leveraging functional magnetic resonance imaging (fMRI) technology, this study aims to comprehensively investigate the brain functional alterations in PD patients exhibiting severe olfactory dysfunction yet maintaining normal cognitive function. By employing a range of indicators from diverse perspectives, we seek to further analyze the correlation between abnormal neuroimaging markers and olfactory scores, ultimately unraveling the potential neuroimaging mechanisms underlying olfactory impairment in PD patients.

## 2 Materials and methods

### 2.1 Participants and data acquisition

In this research, we utilized a publicly accessible dataset sourced from the OpenfMRI database. The accession number is ds000245, and the original article contains comprehensive information regarding both the demography and clinical data (Yoneyama et al., 2018). Patients who were diagnosed with PD at the Department of Neurology at Nagoya University were included in this study. All participants underwent the Japanese Odor Stick Identification Test (OSIT-J) (Saito et al., 2006) and the Addenbrooke’s Cognitive Examination-Revised (ACE-R) (Mioshi et al., 2006) to evaluate their odor-identification ability and cognitive function, respectively. The inclusion criteria were as follows: (1) patients were diagnosed with PD according to the UK Brain Bank criteria (2) patients in this study were between the ages of 55 and 75, had a PD diagnosis after the age of 40, and were in Hoehn and Yahr (HY) Stages I–III. Conversely, a series of stringent exclusion criteria were as follows: (1) the presence of other neurological or psychiatric diseases (2) deep white matter hyperintensity over grade 2 (Fazekas) (3) family history of parkinsonism (4) Addenbrooke’s Cognitive Examination-Revised (ACE-R) score no more than 88. All subjects provided their explicit informed consent by signing the necessary agreement, indicating their willingness to participate in the study. This study received the formal approval of the Ethical Committee of Nagoya University Graduate School of Medicine.

All MRI and functional magnetic resonance imaging (fMRI) scans were executed employing a 3.0T scanner (Siemens, Erlangen, Germany) with a 32-channel head coil at Nagoya University’s Brain and Mind. The high-resolution T1-weighed images were obtained with following standards: repetition time (TR) = 2.5 s, echo time (TE) = 2.48 ms, 192 sagittal slices with 1-mm thickness, field of view (FOV) = 256 mm, matrix = 256 × 256. The resting-state fMRI parameters in this study were carefully controlled to adhere to the following standards: TR = 2.5 s, TE = 30 ms, 39 transverse slices with a 0.5-mm inter-slice interval and 3-mm thickness, FOV = 192 mm, matrix = 64 × 64, flip angle = 80 degrees.

### 2.2 FMRI data preprocessing

Before commencing data analysis, the rs-fMRI data underwent a series of meticulous preprocessing procedures and rigorous quality control measures. The tasks were carried out meticulously using the Data Processing and Analysis of Brain Imaging (DPABI) toolbox (version 6.1),^1^ which is based on MATLAB (The Math Works, Natick, MA, USA) software (Yan et al., 2016). This toolbox was utilized to eliminate the effects of data acquisition, physiological noise, and individual variations in the subject’s brain (Yan et al., 2016). Additionally, it ensured the reliability and sensitivity of the group-level analysis. The preprocessing steps are detailed as follows: (1) The fMRI data originally in DICOM format underwent conversion to 3D-NIFTI format, leveraging the operating environment provided by MATLAB R 2021a. (2) To minimize errors caused by unstable initial environments during image acquisition, we have removed the first 10 time points. (3) The time difference inherent in fMRI images, resulting from compartmentalized scanning, which causes images to be acquired at different times, was rectified. (4) To correct small head movements, head motion correction was utilized. The exclusion thresholds were set at 2 mm and 2°, meaning that any movement exceeding 2 mm in any direction or a rotation exceeding 2° in any direction would be excluded. (5) After undergoing rigorous head motion and time correction, the scanned images were precisely aligned to the MNI space through the application of affine transformation and sophisticated nonlinear registration algorithms. Subsequently, these images were resampled to achieve a uniform resolution of 3 mm × 3 mm × 3 mm. (6) To enhance the signal-to-noise ratio, minimize registration errors, eliminate poorly registered image data, we employ Gaussian smoothing with a 4 mm Full Width Half Maximum (FWHM). (7) The low-frequency band of the BOLD signal offers a more accurate representation of spontaneous neural activities in the human brain during physiological states. Therefore, in this study, we employed high-pass filtering to select the BOLD signal within the frequency range of 0.01–0.1 Hz.

### 2.3 ALFF analysis

Before high-pass filtering was applied, the ALFF was computed using the DPABI software (Yan and Zang, 2010), utilizing the preprocessed dataset. This involved converting the time series data of each individual voxel into a frequency spectrum using the Fourier transform. Then the amplitudes within the frequency range of 0.01–0.1 Hz were summed to calculate the ALFF (Yang et al., 2023; Zuo et al., 2010; Lv et al., 2018). The analysis of variance (ANOVA) was conducted for ALFF in three groups, and the Tukey honest significant difference (HSD) method was used to correct the errors between pairwise comparisons, with age and gender as covariates. A rigorous correction for multiple comparisons was applied at the clump level in this study to minimize the false-positive rates. And the present study then reveals the surviving corrected clumps.

### 2.4 ReHo analysis

We used the DPABI software to perform ReHo analysis based on preprocessed data (prior to smoothing). The ReHo map of the subject was obtained by calculating the Kendall’s coefficient of concordance between the time series of each voxel and the time series of the surrounding 26 voxels in a voxel-wise manner (Choe et al., 2013). Next, the obtained ReHo map still needs to undergo spatial smoothing to improve the signal-to-noise ratio and correct registration errors caused by normalization. The statistical approach for calculating ReHo is identical to the statistical analysis method utilized for calculating ALFF as described earlier. Similarly, we extracted the ReHo values from PD patients.

### 2.5 Complex network analysis

The DPABI NET (version 1.1) toolbox was utilized for brain network construction and subsequent analysis (Yan et al., 2024). The entire brain was partitioned into a total of 116 distinct brain regions using the widely recognized brain atlas, the Anatomical Automatic Labeling (AAL) template (Tzourio-Mazoyer et al., 2002). The Brain Connectivity Toolbox^2^ (Rubinov and Sporns, 2010) was utilized to conduct complex-network analysis, while the BrainNet viewer^3^ was employed to visualize the results (Xia et al., 2013). The analysis of brain network topology encompasses both “small world” properties and node properties. In our study, we calculated the magnitudes of various small-world property across 50 distinct sparsity thresholds. Furthermore, we ascertained the area beneath the curve (AUC) for each small-world parameter within the 0.01–0.34 sparsity interval (Yang et al., 2021), facilitating a more precise identification of changes in the brain’s small-world architecture. Afterward, we calculated the values for the following small-world metrics and node properties at each sparsity level: characteristic shortest path length (L_*p*_), clustering coefficient (C_*p*_), normalized clustering coefficient (γ), normalized characteristic shortest path length (λ), small-worldness (σ), local efficiency (E_*loc*_), global efficiency (E_*glob*_), degree centrality, betweenness centrality (Tzourio-Mazoyer et al., 2002).

### 2.6 Correlation analysis

Using the DPABI software (version 6.1),^1^ we extracted the ALFF, ReHo and FC values for each PD patients (PD patients with severe hyposmia and PD patients with no/mild hyposmia) and subsequently performed a correlation analysis with their OSIT-J scores. During this analysis, we accounted for potential confounding effects by regressing out age and gender as covariates. The statistical significance threshold was established at *P* < 0.005. Furthermore, we performed a correlation analysis to assess the relationship between the brain network indicators of PD patients and OSIT-J scores, with a significance level of *P* < 0.05 being deemed statistically meaningful.

## 3 Results

### 3.1 Demographics and clinical characteristics

In total, 15 PD patients with severe hyposmia (PD-SH) but no cognitive impairment and 15 PD patients with no/mild hyposmia (PD-N/MH) and no cognitive impairment were included in this study. Additionally, 15 healthy subjects without cognitive impairment, hyposmia, and family history of PD were enrolled as healthy controls (HCs). The analysis revealed no statistically significant differences in gender, disease duration, and ACE-R across the PD-SH, PD-N/MH, and HC groups, as depicted in Table 1.

**TABLE 1:** Demographics and clinical characteristics of three groups.

Characteristics	PD		Healthy controls (*n* = 15)	*P*-value
	With severe hyposmia (*n* = 15)	With no/mild hyposmia (*n* = 15)		
Male (*n*%)	7 (46.7%)	6 (40.0%)	7 (46.7%)	NS
Female	8 (53.3%)	9 (60.0%)	8 (53.3%)	NS
Age (years)	70.7 ± 4.8	64.4 ± 7.2	63.3 ± 5.2	0.01
Duration (years)	5.9 ± 3.7	6.1 ± 3.2	–	NS
OSIT-J	1.7 ± 1.1	7.5 ± 1.5	10.4 ± 1.3	*p* < 0.0001
ACE-R	94.3 ± 3.4	96.1 ± 3.1	97.3 ± 2.9	NS
Hoehn–Yahr	2.0 ± 0.4	2.0 ± 0.5	–	NS
**MDS–UPDRS**				
I	5.1 ± 4.2	4.6 ± 3.9	–	NS
II	7.5 ± 4.5	10.3 ± 6.5	–	NS
III	19.3 ± 7.9	21.2 ± 9.4	–	NS
IV	1.9 ± 3.5	2.0 ± 3.3	–	NS

Data are means ± standard deviation (SD). PD, Parkinson’s disease; OSIT-J, Odor Stick Identification Test for the Japanese; ACE-R, Addenbrooke’s Cognitive Examination-Revised; MDS-UPDRS, Movement Disorder Society-Sponsored Revision of the Unified Parkinson’s Disease Rating Scale; NS, not significantly different; The gender differences were analyzed using the chi-square test. For the comparison of Hoehn and Yahr stages and MDS-UPDRS-IV scores, the Student’s *t*-test was employed. To assess differences in disease duration and MDS-UPDRS-I/II/III scores, the Mann–Whitney U test was utilized. Additionally, age at examination and ACE-R scores were analyzed using one-way analysis of variance (ANOVA). Lastly, the Kruskal–Wallis test was applied to evaluate differences in OSIT-J scores.

### 3.2 Results of ALFF analysis

#### 3.2.1 PD-SH V.S. HC

**FIGURE 1:**
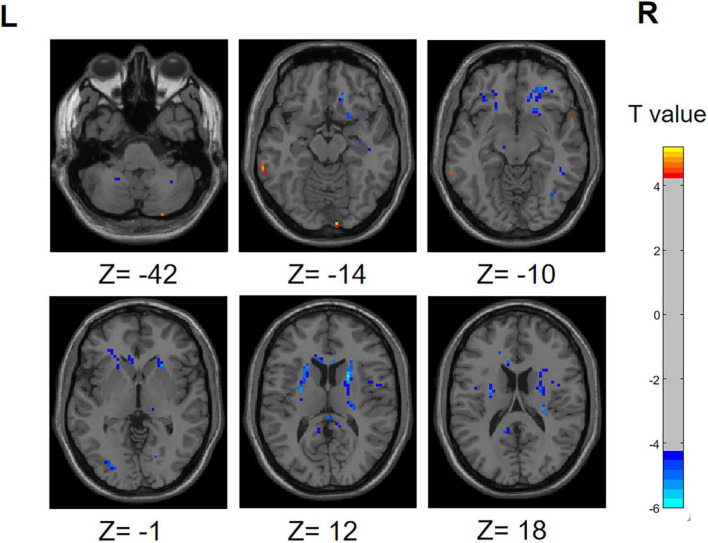
Differences in ALFF between PD-SH group and HC group. The color red denotes increased ALFF in PD-SH group compared to HC group, while color blue denotes decreased ALFF in PD-SH group compared to HC group. The corresponding color bar denotes the *t*-value. R, right; L, left.

The PD-SH group showed that the ALFF value of right inferior cerebellum (AAL: Cerebelum_Crus2_R), left inferior temporal gyrus, left middle temporal gyrus and right temporal pole: superior temporal gyrus was significantly higher (FDR correction method with a significance threshold of *P* < 0.05) than those of the HC group (Figure 1). The ALFF of PD-SH group in bilateral putamen, right rolandic operculum, right insula, bilateral precuneus, right posterior cingulate gyrus, right superior frontal gyrus, orbital part, right gyrus rectus, right olfactory cortex, right hippocampus, bilateral cerebellum (AAL: Cerebelum_8), right inferior occipital gyrus, right inferior frontal gyrus, orbital par, left middle frontal gyrus, orbital part, right lingual, right thalamus, left posterior cingulate gyrus and right rolandic operculum is lower than HC group (Figure 1).

#### 3.2.2 PD-N/MH V.S. HC

Compared with the HCs, the ALFF in the bilateral temporal pole: superior temporal gyrus of the PD-N/MH group significantly increased (FDR correction, *P* < 0.01). As shown in Supplementary Figure 1, the ALFF of PD-N/MH group in the left middle occipital gyrus, right putamen, right caudate, left posterior cingulate gyrus and right median cingulate and paracingulate gyri are lower than HC group (FDR correction, *P* < 0.01).

#### 3.2.3 PD-N/MH V.S. PD-SH

Illustrated in Supplementary Figure 2, the PD-SH exhibited a noteworthy enhancement in ALFF within bilateral superior temporal gyrus, bilateral middle temporal gyrus, left superior cerebellum, left superior frontal gyrus, dorsolateral and inferior frontal gyrus, triangular part, when compared to the PD-N/MH group (voxel *p* < 0.001, cluster *p*-value < 0.05, GFR corrected). The ALFF of PD-SH group in left inferior cerebellum, vermis and left anterior cingulate and paracingulate gyri are lower than PD-N/MH group (voxel *p* < 0.001, cluster *p*-value < 0.05, GFR corrected).

### 3.3 Results of ReHo analysis

#### 3.3.1 PD-SH V.S. HC

**FIGURE 2:**
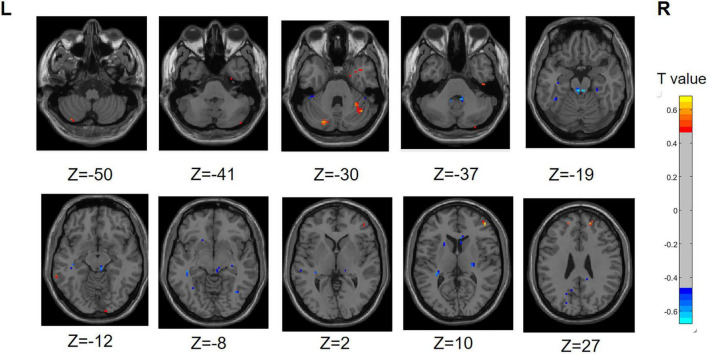
Differences in ReHo between PD-SH group and HC group. The color red denotes increased ReHo in PD-SH group compared to HC group, while color blue denotes decreased ReHo in PD-SH group compared to HC group. The corresponding color bar denotes the *t*-value. R, right; L, left.

In the PD-SH group, a statistically significant elevation (FDR correction method with a significance threshold of *P* < 0.05) of ReHo was observed in region of left Inferior Cerebellum, left Middle temporal gyrus, right Lingual gyrus, right Inferior frontal gyrus, triangular part, right Middle frontal gyrus, and bilateral Superior frontal gyrus, dorsolateral (Figure 2), when compared to the HC group. In comparison to the HC group, the PD-SH group demonstrated decreased (FDR correction, *P* < 0.05) ReHo in specific regions, including (AAL: Cerebelum_9), bilateral Fusiform gyrus, bilateral Hippocampus, left Middle temporal gyrus, left Inferior temporal gyrus, right Inferior occipital gyrus, left Superior occipital gyrus, right Thalamus, left Precuneus, left Cuneus, right Caudate nucleus and left Lenticular nucleus, putamen.

#### 3.3.2 PD-N/MH V.S. HC

The ReHo values in region of bilateral Inferior Cerebellum (AAL: Cerebelum_Crus2), right Superior frontal gyrus, medial orbital, and right Superior frontal gyrus, dorsolateral were significantly higher (FDR correction, *P* < 0.05) in the PD-N/MH group compared to the HC group. Additionally, in the PD-NMH group, lower (FDR correction, *P* < 0.05) ReHo values were observed in regions right Fusiform gyrus, left Superior temporal gyrus, left Lenticular nucleus, putamen, bilateral Posterior cingulate gyrus, right Caudate nucleus and left Precental gyrus compared to the HC group (Supplementary Figure 3).

#### 3.3.3 PD-N/MH V.S. PD-SH

**FIGURE 3:**
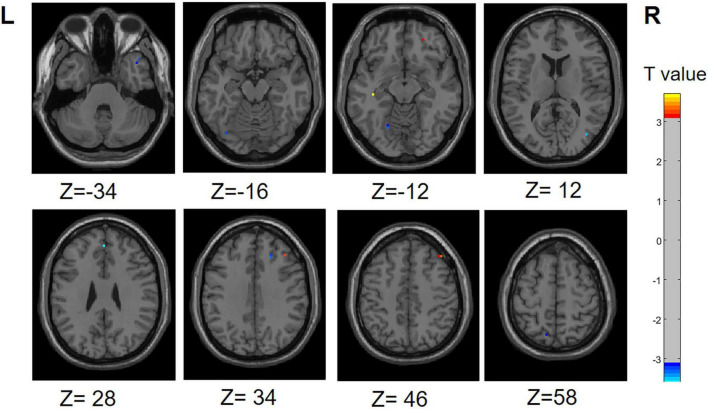
Differences in ReHo between PD-N/MH group and PD-SH group. The color red denotes increased ReHo in PD-N/MH group compared to PD-SH group, while color blue denotes decreased ReHo in PD-N/MH group compared to PD-SH group. The corresponding color bar denotes the *t*-value. R, right; L, left.

Compared to the PD-SH group, the PD-N/MH group exhibited decreased (voxel level *p* < 0.001, cluster *p*-value < 0.05, GFR corrected) ReHo in the following regions, including the left Precuneus, left fusiform, left Superior Cerebellum, left Superior frontal gyrus, medial, right Superior frontal gyrus, and right Middle occipital gyrus. Furthermore, our findings revealed that PD-N/MH patients displayed a notable enhancement (voxel *p* < 0.001, cluster *p*-value < 0.05, GFR corrected) in ReHo within the right Middle frontal gyrus, left Hippocampus and right Superior frontal gyrus, orbital part in comparison to PD-SH patients (Figure 3).

### 3.4 Results of functional connectivity analysis

#### 3.4.1 PD-SH V.S. HC

**FIGURE 4:**
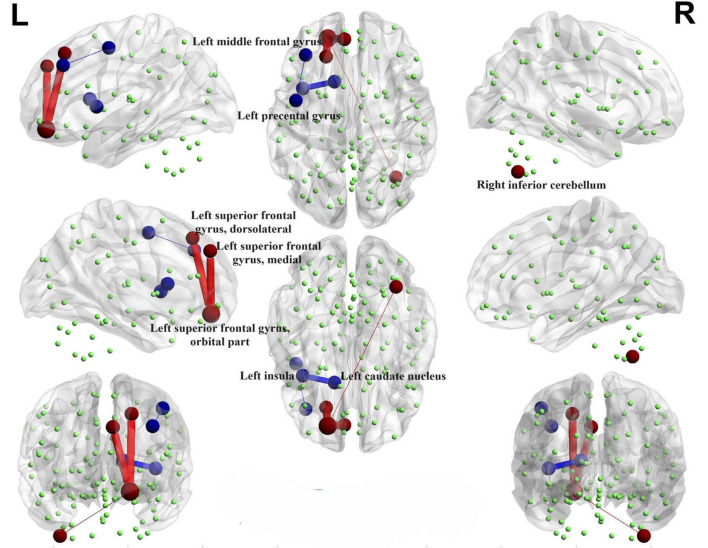
Functional connectivity alterations between the PD-SH group and the HC group. The spheres represent brain nodes, where red spheres and connecting lines signify strengthened functional connectivity, while blue indicates a decrease in functional connectivity. R, right; L, left.

Illustrated in Figure 4, We observed that the PD-SH group exhibited decreased connectivity in specific regions (FDR correction, *P* < 0.00001), including the connection between the left middle frontal gyrus and left precental gyrus, as well as the link between the left insula and left caudate nucleus, when compared to the HC group. Furthermore, there are regions where connectivity is notably strengthened, including the connection between the left superior frontal gyrus, dorsolateral and left superior frontal gyrus, orbital part, the connection between the left superior frontal gyrus, medial and the left superior frontal gyrus, orbital part, as well as the connection between the right inferior cerebellum and the left superior frontal gyrus, orbital part (FDR correction, *P* < 0.00001).

#### 3.4.2 PD-N/MH V.S. HC

**FIGURE 5:**
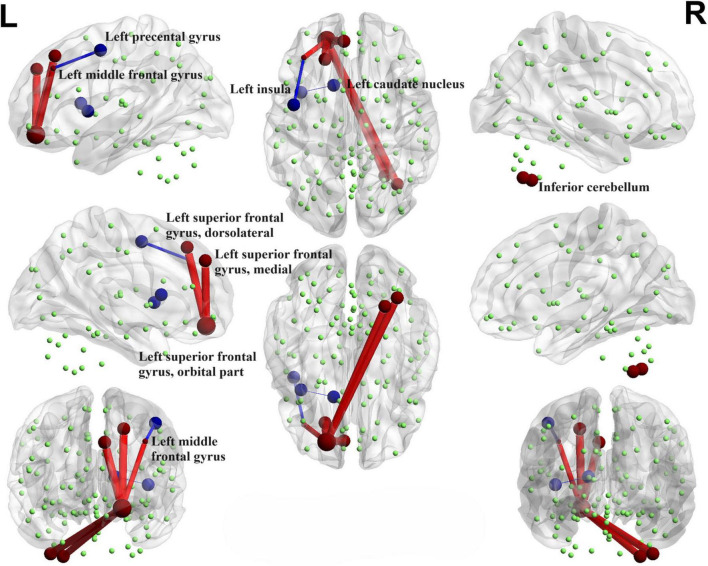
Functional connectivity alterations between the PD-N/MH group and the HC group. The spheres represent brain nodes, where red spheres and connecting lines signify strengthened functional connectivity, while blue indicates a decrease in functional connectivity. R, right; L, left.

Interestingly, when comparing the PD-N/MH group to the HC group, we also observed similarly decreased functional connectivity regions (FDR correction, *P* < 0.00001), particularly in the connections between the left middle frontal gyrus and the left precental gyrus, and connections between the left insula and left caudate nucleus (Figure 5). In addition, when compared to the HC group, the PD-N/MH group demonstrated regions with notably increased connectivity, specifically in the connections between the left superior frontal gyrus, dorsolateral and left superior frontal gyrus, orbital par, the left superior frontal gyrus, medial and the left superior frontal gyrus, orbital part, the left middle frontal gyrus and the left superior frontal gyrus, orbital part, as well as the left superior frontal gyrus, orbital part and the right inferior cerebellum (Cerebelum_7b_R, Cerebelum_8_R) (FDR correction, *P* < 0.00001).

#### 3.4.3 PD-SH V.S. PD-N/MH

**FIGURE 6:**
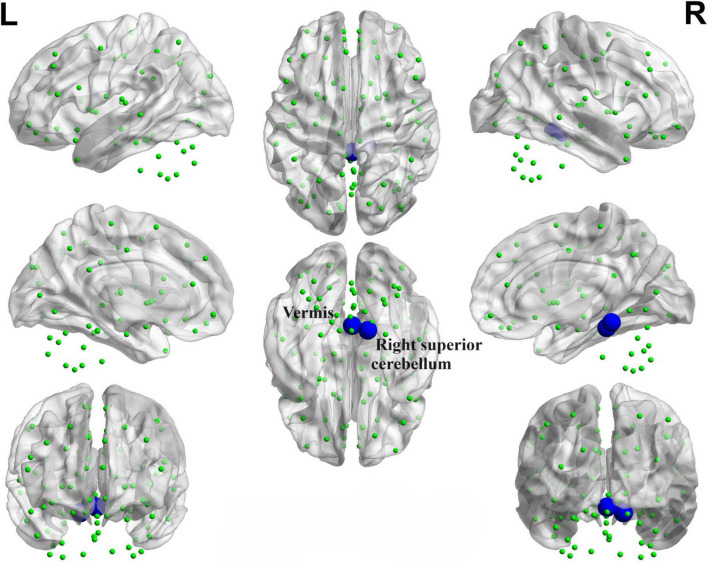
Decreased functional connectivity between the right superior cerebellum and the vermis of the PD-SH group in comparison to the PD-N/MH group. The blue spheres and connecting lines signify decreased functional connectivity. R, right; L, left.

We conducted a comparison of brain functional connectivity strength between the PD-SH group and the PD-N/MH group (Figure 6), revealing that the PD-SH group demonstrated significantly decreased connectivity between the right superior cerebellum and the vermis (*P* < 0.05).

### 3.5 Results of complex network analysis

#### 3.5.1 Small-worldness

Our findings indicated alterations in the topological properties of brain functional networks among PD-SH patients. Nevertheless, there was no statistically significant difference in the small world properties of brain networks between the PD-SH and PD-N/MH group.

**FIGURE 7:**
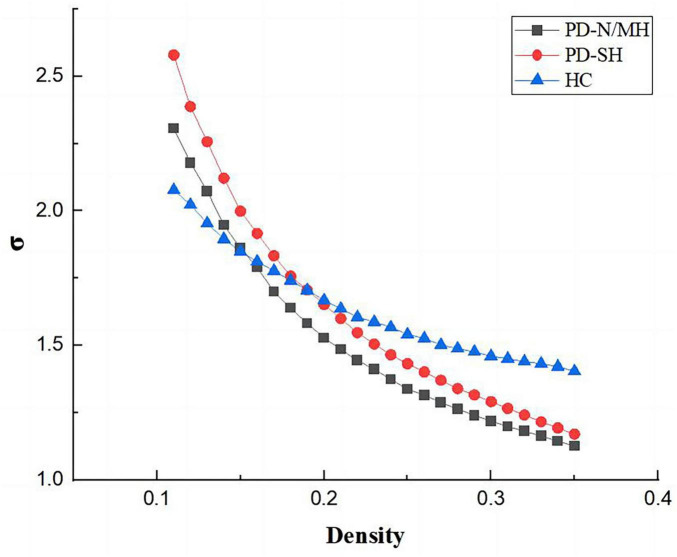
Comparison of “Small World” property among three groups.

**FIGURE 8:**
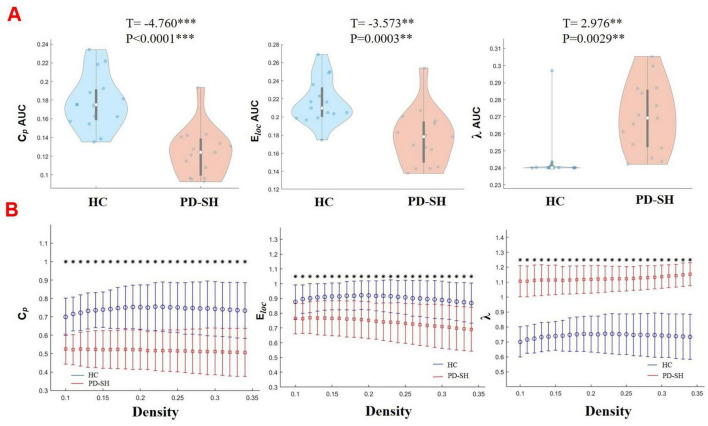
Differences in network topological properties among the PD-SH, PD-N/MH, and HC groups. **(A)** Violin plots depict the distribution of mean Cp AUC, Eloc AUC and λ values, highlighting the contrast between PD-SH and HC. **(B)** Cp, Eloc and λ values are shown across a density range spanning from 10 to 34%. Each point, accompanied by an error bar, represents the mean and standard deviation at specific density levels, respectively. The asterisk indicates the significant difference levels. ** denotes *P* < 0.01; *** denotes *P* < 0.0001.

As shown in the Figures 7, 8, the PD-N/MH, PD-SH, and HC groups all exhibited a sigma (σ) value greater (FDR correction, *P* < 0.05) than 1 and a lambda (λ) value close to 1, it can be inferred that both the PD and HC groups possess small-world properties in their brain networks (Yan et al., 2013). Compared to the HC group, the PD-SH group exhibited significantly reduced values (FDR correction, *P* < 0.05) of Cp and Eloc, while the lambda value was significantly elevated (Figure 8).

Similarly, the PD-N/MH group also demonstrated significantly lower values of Cp and Eloc compared to the HC group, along with higher λ values (Supplementary Figure 4).

#### 3.5.2 Nodal properties

**FIGURE 9:**
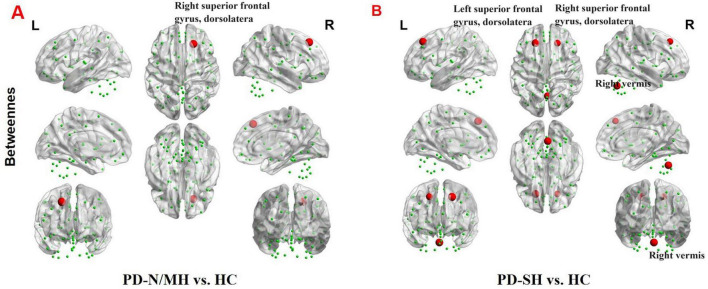
The brain regions with statistically significant difference in betweenness centrality among PD-SH, PD-N/MH an HC group. **(A)** The difference in betweenness centrality between the PD-N/MH and HC group. **(B)** The difference in betweenness centrality between the PD-SH and HC group. The red spheres represent the brain nodes with increased betweenness centrality in PD-N/MH or PD-SH compared to HC group. R, right; L, left.

Significant disparities were observed in the nodal characteristics between the PD group and the HC group, as detailed below: In comparison to the HC group, patients with PD-SH demonstrated a notable elevation (FDR correction, *P* < 0.05) in the nodal betweenness centrality of the bilateral superior frontal gyrus, dorsolateral and right vermis; the betweenness centrality of the node located in the right superior frontal gyrus, dorsolateral was notably elevated (FDR correction, *P* < 0.05) in the PD-N/MH group compared to the HC group (Figure 9).

**FIGURE 10:**
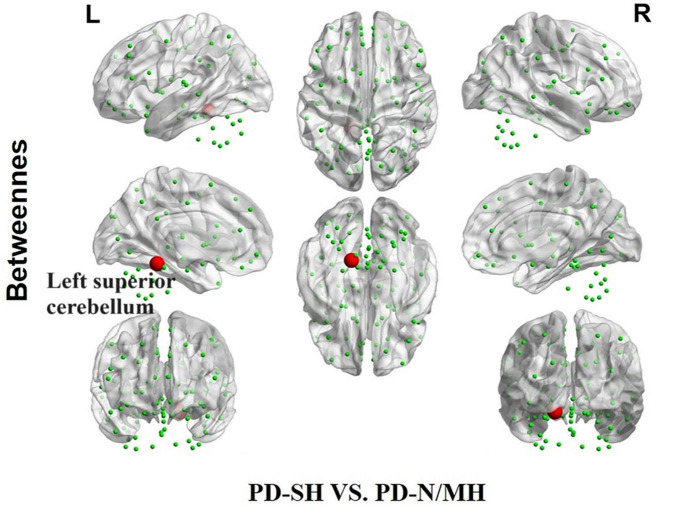
The difference in betweenness centrality between the PD-SH and PD-N/MH group. The red spheres represent the brain nodes with increased betweenness centrality in PD-N/MH compared to PD-SH group. R, right; L, left.

Compared to the PD-SH group, the PD-N/MH group demonstrated a notable increase (*P* < 0.05) in betweenness centrality within the left superior cerebellum (Figure 10).

**FIGURE 11:**
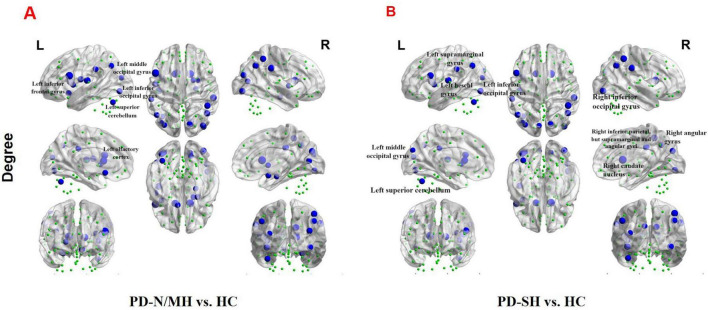
The brain regions with statistically significant difference in nodal degree among PD-SH, PD-N/MH an HC group. **(A)** The difference in nodal degree between the PD-N/MH and HC group. **(B)** The difference in nodal degree between the PD-SH and HC group. The blue spheres represent the brain nodes with increased nodal degree in PD-N/MH or PD-SH compared to HC group. R, right; L, left.

We further observed a significant decrease (FDR correction, *P* < 0.05) in nodal degree in multiple brain regions of PD-SH patients compared to the HC group, specifically the left inferior frontal gyrus, opercular part, left insula, bilateral cuneus, left middle occipital gyrus, bilateral inferior occipital gyrus, right inferior parietal, but supramarginal and angular gyri, bilateral supramarginal gyrus, bilateral angular gyrus, bilateral caudate nucleus, left heschl gyrus and left superior cerebellum (Figure 11).

Compared to the HC group, the PD-N/MH group also exhibited regions with significantly reduced (FDR correction, *P* < 0.05) nodal degree, as follows (Figure 11): Left inferior frontal gyrus, opercular part, left rolandic operculum, left olfactory cortex, left insula, right parahippocampal gyrus, right amygdala, right calcarine fissure and surrounding cortex, right cuneus, bilateral middle occipital gyrus, bilateral inferior occipital gyrus, right inferior parietal, but supramarginal and angular gyri, bilateral supramarginal gyrus, bilateral angular gyrus, bilateral caudate nucleus, right lenticular nucleus, pallidum and left superior cerebellum.

We found that compared with PD-SH patients, PD-N/MH patients exhibited a significant decrease (*P* < 0.05) in nodal degree in the left superior temporal lobe (Supplementary Figure 5).

### 3.6 Results of correlation analysis

**FIGURE 12:**
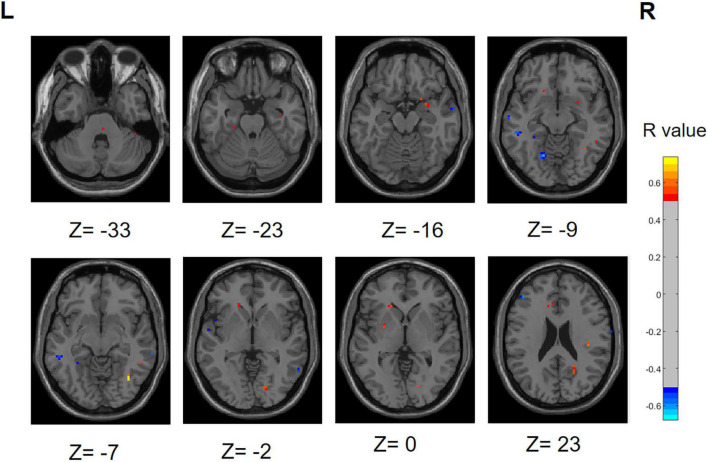
Correlation maps illustrating the relationship between ALFF values and olfactory score in PD patients. Warm colors, such as red, denote a positive correlation, while cool colors, like blue, signify a negative correlation. The color bar denotes the *R*-value. R, right; L, left.

We performed a correlation analysis to investigate the relationship between the ALFF values of patients in the PD group (PD-SH group and PD-N/MH group) and their olfactory scores (OSIT-J score), and the findings are presented below (Figure 12): the ALFF value observed in bilateral superior cerebellum, bilateral fusiform gyrus, right lingual gyrus, bilateral middle temporal gyrus, left inferior frontal gyrus, triangular part and bilateral supramarginal gyrus demonstrated a statistically significant positive correlation with the olfactory scores (*R* > 0.5). Conversely, the ALFF values in the bilateral middle temporal gyrus, left lingual gyrus, left superior temporal gyrus, left temporal pole: superior temporal gyrus, right inferior temporal gyrus, right postcentral gyrus, left middle frontal gyrus displayed a negative correlation with olfactory scores.

**FIGURE 13:**
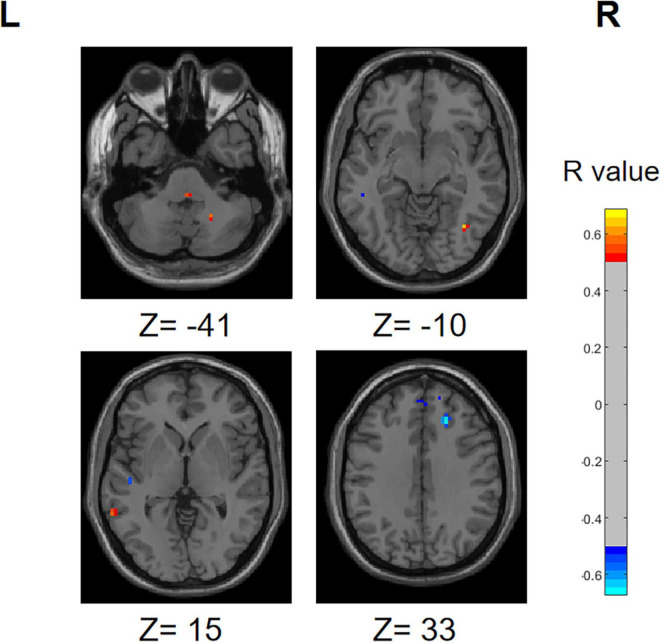
Correlation maps illustrating the relationship between ReHo values and olfactory score in PD patients. Warm colors, such as red, denote a positive correlation, while cool colors, like blue, signify a negative correlation. The color bar denotes the *R*-value. R, right; L, left.

Similarly, we performed a correlation analysis to assess the relationship between the ReHo values and olfactory scores among patients with PD (Figure 13). The following regions demonstrated a positive correlation with olfactory scores: right inferior cerebellum, left inferior temporal gyrus, left fusiform gyrus, right inferior occipital gyrus, right superior temporal gyrus and right middle temporal gyrus (*R* > 0.5). Conversely, the following regions right middle frontal gyrus, right superior frontal gyrus, right superior frontal gyrus, medial and right middle occipital gyrus displayed a negative correlation with olfactory scores.

**FIGURE 14:**
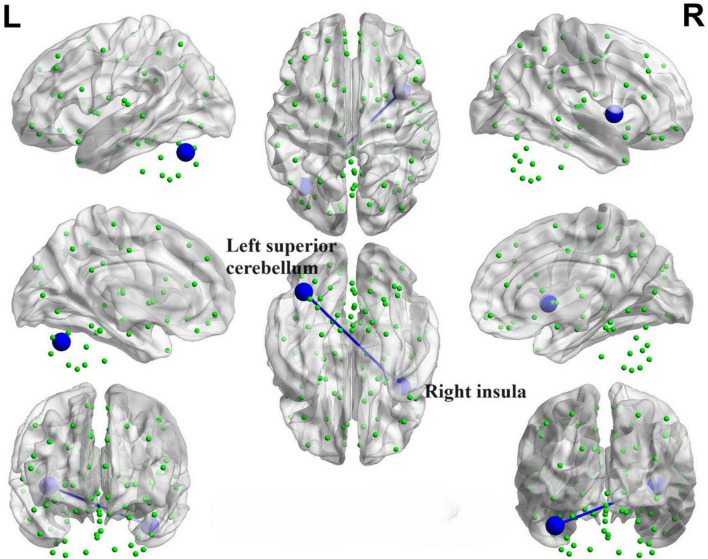
Brain map depicting functional connectivity with negative correlation. The azure sphere serves as a symbol, representing a node that corresponds to a distinct brain region, whereas the blue line denotes the existence of a negative correlation between functional connectivity and olfactory score. R, right; L, left.

During the correlation analysis between FC values and OSIT-J scores, a negative correlation (*P* < 0.005) was observed between olfactory scores and the functional connectivity between the left superior cerebellum and right insula (Figure 14).

**FIGURE 15:**
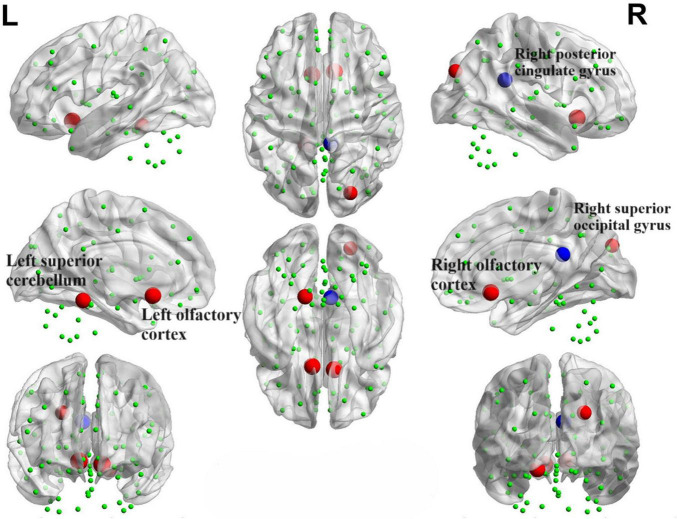
Brain map portraying nodes exhibiting correlations with OSIT-J scores. The red sphere signifies a positive correlation between the nodal betweenness and the olfactory score, whereas the blue sphere denotes a negative correlation.

We identified positive correlations between the nodal betweenness and olfactory scores in several brain regions (Figure 15), particularly the bilateral olfactory cortex, right superior occipital gyrus and left superior cerebellum (*P* < 0.05). In addition, the nodal betweenness of right posterior cingulate gyrus was found to be negatively correlated with olfactory scores.

However, upon analysis, it was determined that there was no significant correlation between node degree, small-world property values, and olfactory scores.

## 4 Discussion

### 4.1 Relationship between functional separation and hyposmia

Functional separation serves as a tool to assess the local functional properties of brain regions, encompassing two key indicators: ALFF and ReHo (Choe et al., 2013; Yu et al., 2021). ALFF serves as a measure of spontaneous neural activity within local brain regions. Elevated ALFF values indicate an increase in spontaneous neural activity, whereas lower ALFF values suggest a decrease in such activity (Zou et al., 2008). ReHo, an indicator of the synchronicity of neural activity within local brain regions (Zhang et al., 2017). In this study, we observed the existence of differential brain regions in both the PD-SH and PD-N/MH groups when compared to the ALFF values in the HC group. Notably, the PD-SH group exhibited a greater number of differential brain regions compared to the PD-N/MH group. We observed a significant elevation in ALFF values within the superior temporal gyrus in both the PD-SH and PD-N/MH groups, compared to the HC group. Furthermore, the ALFF values in the superior temporal gyrus were notably elevated in the PD-SH group in comparison to the PD-NMH group. Given the observed negative correlation between ALFF values in the superior temporal gyrus and olfactory scores, we hypothesize that the superior temporal gyrus plays a role in the pathophysiology of olfactory dysfunction in patients with PD.

In patients with Parkinson’s disease (PD), the ReHo values in the inferior cerebellum are elevated compared to those in the healthy control (HC) group. Additionally, a positive correlation exists between the ReHo values in the inferior cerebellum and olfactory scores, hinting at a potential decrease in neuronal activity within this region during olfactory dysfunction. However, the magnitude of this decrease may be modest, as no significant differences in ReHo values were detected between the PD-SH group and the PD-N/MH group in the inferior cerebellum.

The ReHo values observed in the middle temporal gyrus and superior frontal gyrus of patients with Parkinson’s disease (PD) are notably elevated compared to those in the healthy control (HC) group. Furthermore, the PD-SH group, who exhibit severe olfactory dysfunction, demonstrate significantly higher ReHo values in the middle temporal gyrus and superior frontal gyrus than the PD-N/MH group. This indicates a compensatory enhancement of neuronal activity in these regions among PD patients with severe olfactory impairment. This finding is corroborated by the negative correlation between ReHo values in the superior frontal gyrus and olfactory scores.

Significant differences in ReHo values are observed in the occipital middle gyrus between the PD-SH and PD-N/MH groups, with a negative correlation evident between ReHo values in this region and olfactory scores. Previous research has unequivocally demonstrated that PD patients afflicted with visuospatial impairment exhibit a more severe impairment of olfactory function compared to their counterparts (Cecchini et al., 2019). This observation underscores the likelihood of intricate and extensive cross-connections and interactive mechanisms operating among diverse sensory systems. Moreover, numerous in-depth studies have illuminated the profound interplay between olfaction and vision (Boot et al., 2024; Stickel et al., 2019; Sijben et al., 2018). Specifically, olfactory cues can reflexively steer the direction of visual attention, while visual stimuli, in turn, can modulate an individual’s perception and identification of odors. Notably, there is a tight coupling between the cross-modal integration of visual and olfactory information and the activation patterns within the occipital cortex of the brain (Ripp et al., 2018). Drawing upon this insight, and integrating our current research findings, we can formulate a plausible hypothesis that the compensatory augmentation of local neuronal activity within the occipital lobe in PD patients may be associated with olfactory dysfunction. This hypothesis not only enhances our understanding of the pathophysiological intricacies of PD but also opens up novel vistas and avenues for the development of therapeutic interventions aimed at ameliorating olfactory and other sensory deficits in PD patients.

Research has demonstrated notable gray matter atrophy in the frontal, temporal, and occipital regions among patients with Parkinson’s disease (PD) (Ren et al., 2023). Nevertheless, our study revealed that PD patients experiencing severe olfactory dysfunction exhibited heightened brain functional activity in certain areas within these regions. This observation hints at a potential role of the frontal, temporal, and occipital regions in olfactory dysfunction among PD patients. Notably, this discovery is in congruence with prior research outcomes that have established a link between olfactory function and executive abilities (Solla et al., 2023). More precisely, the underlying mechanisms for this regional activation may encompass intricate neural interactions spanning multiple levels, along with compensatory mechanisms that come into play. However, further investigation is warranted to elucidate the specific molecular mechanisms involved and their interplay with neurotransmitters, including dopamine, serotonin, and acetylcholine.

### 4.2 Relationship between functional connectivity and hyposmia

We observed that, in comparison to the HC group, the PD-SH and PD-N/MH groups exhibited weaker connections in the left middle frontal gyrus, the left precental gyrus, as well as between the left insula and the left caudate nucleus. Furthermore, the PD patients in both groups exhibited stronger functional connectivity compared to the HC group in the following regions: the dorsolateral and orbital parts of the left superior frontal gyrus, the connection between the medial and orbital parts of the left superior frontal gyrus, and the connection between the right inferior cerebellum and the orbital part of the left superior frontal gyrus. Based on previous research on Parkinson’s disease (PD), the accumulation of Lewy bodies in the prefrontal cortex and the disruption of the basal ganglia motor circuit represent the characteristic pathological changes associated with the condition (Wichmann, 2019). Therefore, our findings indicate that the aberrant functional connectivity observed in the aforementioned regions is likely associated with PD itself, rather than being a consequence of olfactory dysfunction related alterations. It is worth noting that compared to the PD-N/MH group, the PD-SH group displayed significant abnormalities in functional connectivity within the cerebellum. Interestingly, the functional connectivity between the superior cerebellar lobule and the insula showed a negative correlation with olfactory scores, indicating that compensatory enhancement of cerebellar functional connectivity occurs in PD patients with olfactory dysfunction. This finding aligns with our prior research, revealing that the cerebellum and insula are linked to olfactory function in PD patients and exhibit aberrant functional connectivity with white matter fiber bundles (Wang et al., 2022; Du et al., 2023). Consequently, our study reinforces the potential significance of the cerebellum and insula in olfactory impairments among PD patients.

### 4.3 Alterations in network topology

In this study, using graph-theoretical analysis methods, we explored the alterations in the topological properties of brain networks in patients with PD who also had severe olfactory dysfunction. Some previous researches on the topological attributes of brain networks in PD patients have consistently shown the maintenance of small-world properties, which agrees with our own findings (Guo et al., 2018; Suo et al., 2017; Hou et al., 2018). Our investigation has shown that both PD-SH and PD-N/MH possess small-world characteristics within their functional brain networks, nonetheless, notable changes have occurred in the topological attributes of these networks. When comparing PD patients with HC, it is observed that PD patients exhibit a lower clustering coefficient and local efficiency, coupled with a higher normalized characteristic shortest path length. These findings indicate that the topological structure of the brain networks in PD patients was disrupted, resulting in decreased efficiency in local information processing and integration, and a shift toward a more randomized functional brain network (Luo et al., 2015). Certain studies have demonstrated a strong correlation between olfactory dysfunction and both local and global efficiency (Park et al., 2019). Perhaps the olfactory dysfunction arises from the decreased local efficiency of the brain network, which impairs the transmission of olfactory information. Despite our efforts, we did not detect any significant differences in the small-world properties of the brain between the PD-SH and PD-N/MH groups. Based on this observation, we hypothesize that the impact of olfactory dysfunction on the brain’s small-world attributes in PD patients is rather minimal, to the extent that it cannot be reliably detected in smaller sample sizes.

During the analysis of node attributes, we found significant differences in node betweenness centrality between the PD-SH and PD-NMH groups compared to the HC group. Notably, the PD-SH group exhibited a larger number of nodes with such differences. Additionally, the PD-NMH group showed a significant increase in nodal betweenness centrality specifically in the superior cerebellar lobule compared to the PD-SH group. Intriguingly, a positive correlation was observed between the betweenness centrality of the superior cerebellar lobule and olfactory scores. These observations strongly suggest that a decrease in betweenness centrality in the superior cerebellar lobule may contribute to the pathogenesis of olfactory dysfunction.

Significant differences in node degree within the superior temporal gyrus have been observed between the PD-SH and PD-N/MH groups. Despite the absence of a significant correlation between these node degree differences and olfactory scores, we cannot conclusively dismiss the potential role of the superior temporal gyrus in olfactory dysfunction. This lack of correlation may be attributed to statistical errors arising from a limited sample size.

### 4.4 Limitations

Firstly, the data utilized in this study was sourced from public databases. However, in the future, it is imperative that we personally gather data to enhance its precision and reliability. Secondly, this study adopts a cross-sectional and retrospective approach, which poses a challenge in determining whether olfactory dysfunction precedes changes in brain function imaging or whether modifications in brain function precede the observed imaging manifestations. Consequently, it is imperative to conduct longitudinal and prospective studies in the future, encompassing follow-up research on PD-N/MH patients, to further clarify these relationships. Thirdly, the small sample size of this study represents a limitation that may impact the validity, reliability, and applicability of our research findings. Consequently, we emphasize the importance of conducting future studies with larger sample sizes. Finally, it is noteworthy that task-based functional magnetic resonance imaging (fMRI) exhibits enhanced sensitivity and accuracy in detecting brain function changes. Consequently, in the future, it is advisable to consider utilizing fMRI examinations that involve olfactory stimulation to delve deeper into the mechanisms responsible for olfactory dysfunction in PD patients.

## 5 Conclusion

Utilizing indices such as ALFF, ReHo, FC, and brain network topological properties, this study revealed significant differences in brain function among patients categorized into the PD-SH, PD-N/MH, and HC groups, specifically with regards to their brains’ functional segregation and integration capabilities.

From the lens of local brain function, we speculate that alterations in the functional activities within the temporal, frontal, occipital lobes, and the cerebellum contribute significantly to the olfactory impairment observed in patients with Parkinson’s disease. From the angle of brain functional connectivity, the presence of abnormal connectivity between the cerebellum and the insula, as well as internally within the cerebellum, could potentially contribute to the olfactory impairment observed in patients with Parkinson’s disease. Additionally, in terms of brain network topological properties, despite sharing small-world properties, notable differences in node degree within the superior temporal gyrus and the betweenness centrality of the superior cerebellar lobule between the PD-SH and PD-N/MH groups indicate potential associations between alterations in nodal characteristics of these regions and olfactory dysfunction. Based on these comprehensive analyses, we hypothesize that these regions play a crucial role in the pathogenesis of olfactory dysfunction observed in PD patients.

## Data Availability

Publicly available datasets were analyzed in this study. This data can be found here: https://openfmri.org/dataset/ds000245/ with its accession number ds000245.

## Footnotes

1
http://rfmri.org/dpabi

2
https://www.nitrc.org/projects/bct/

3
https://www.nitrc.org/projects/bnv/
