# Supplementary material for: Functional activity, functional connectivity and complex network biomarkers of progressive hyposmia Parkinson’s disease with no cognitive impairment: evidences from resting-state fMRI study
Source: Front Aging Neurosci. 2024 Sep 25;16:1455020. doi: 10.3389/fnagi.2024.1455020 (PMC11461260; doi:10.3389/fnagi.2024.1455020)
Supplement: Supplementary file 1 [file Data_Sheet_1.PDF]

Supplementary Material

1 Supplementary Figures and Tables

1.1 Supplementary Figures

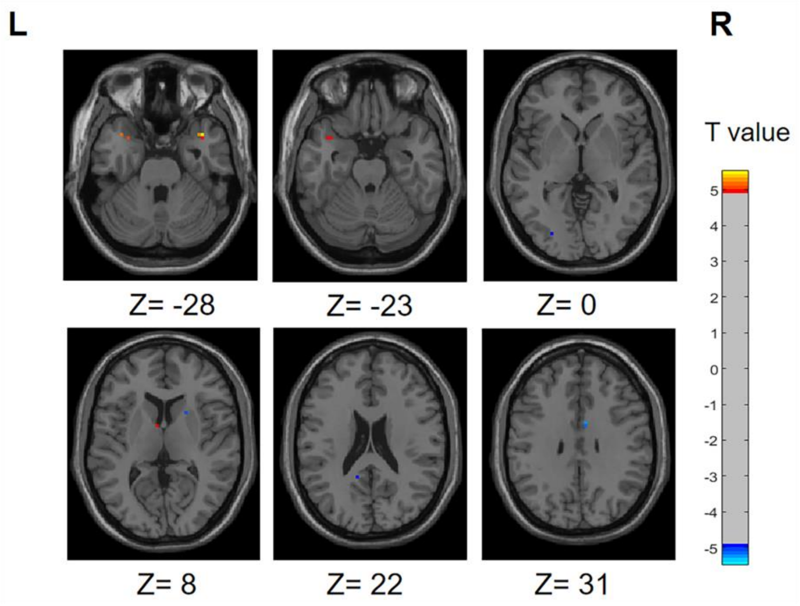

Supplementary Figure 1. Differences in ALFF between PD-N/MH group and HC group. The color red denotes increased ALFF in PD-N/MH group compared to HC group, while color blue denotes decreased ALFF in PD-N/MH group compared to HC group. The corresponding color bar denotes the t-value. R: right; L: left.

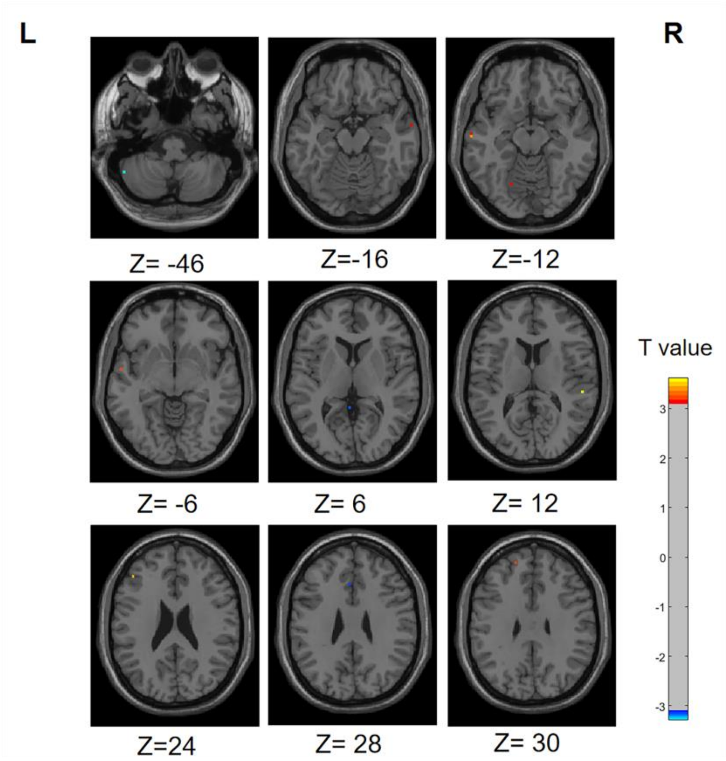

Supplementary Figure 2. Differences in ALFF between PD-N/MH group and PD-SH group. The color red denotes increased ALFF in PD-SH group compared to PD-N/MH group, while color blue denotes decreased ALFF in PD-SH group compared to PD-N/MH group. The corresponding color bar denotes the t-value. R: right; L: left.

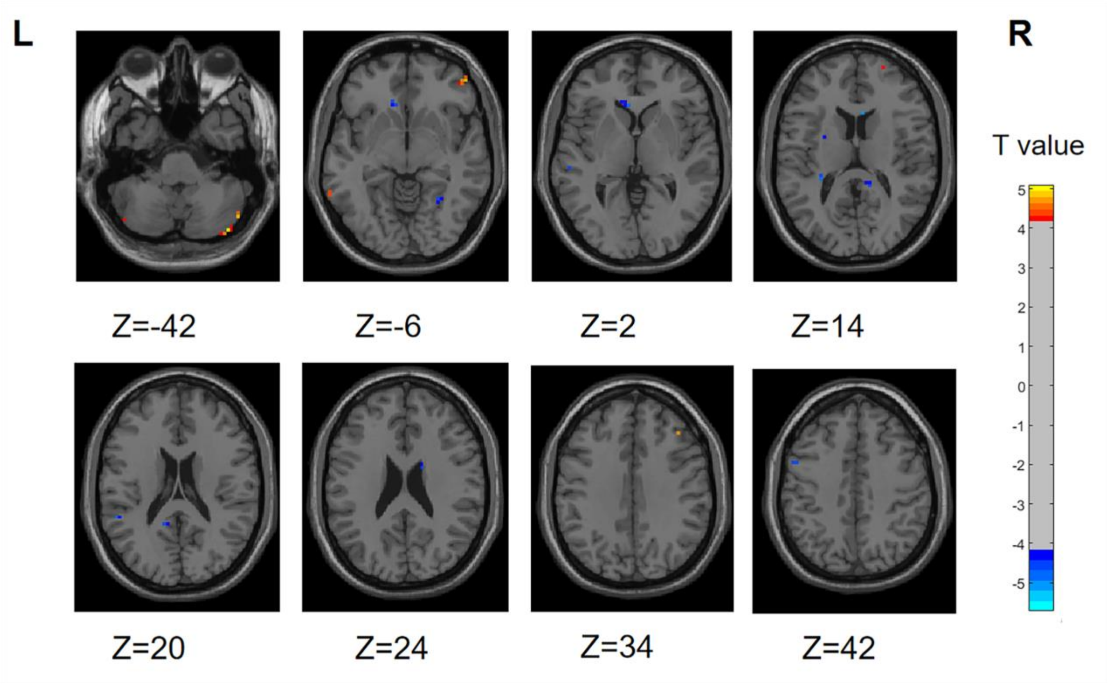

Supplementary Figure 3. Differences in ReHo between PD-N/MH group and HC group. The color red denotes increased ReHo in PD-N/MH group compared to HC group, while color blue denotes decreased ReHo in PD-N/MH group compared to HC group. The corresponding color bar denotes the t-value. R: right; L: left.

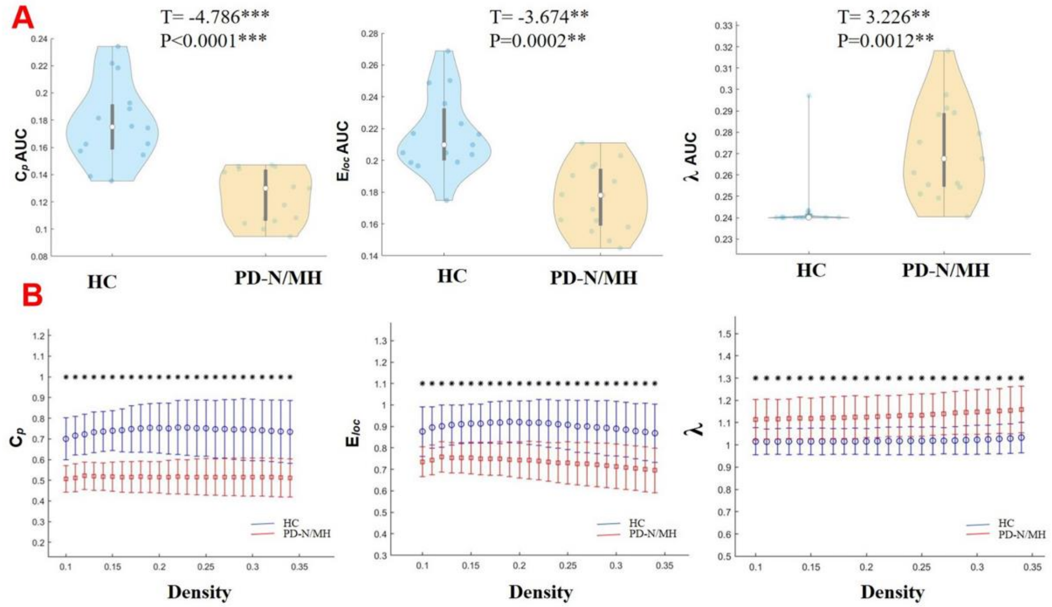

Supplementary Figure 4. Differences in network topological properties between the HC and PD-N/MHgroups. **(A)**: Violin plots depict the distribution of mean Cp AUC, Eloc AUC and λ values, highlighting the contrast between PD-N/MH and HC. **(B)**: Cp, Eloc and λvalues are shown across a density range spanning from 10% to 34%. Each point, accompanied by an error bar, represents the mean and standard deviation at specific density levels, respectively. ** denotes P <0.01; *** denotes P <0.0001.

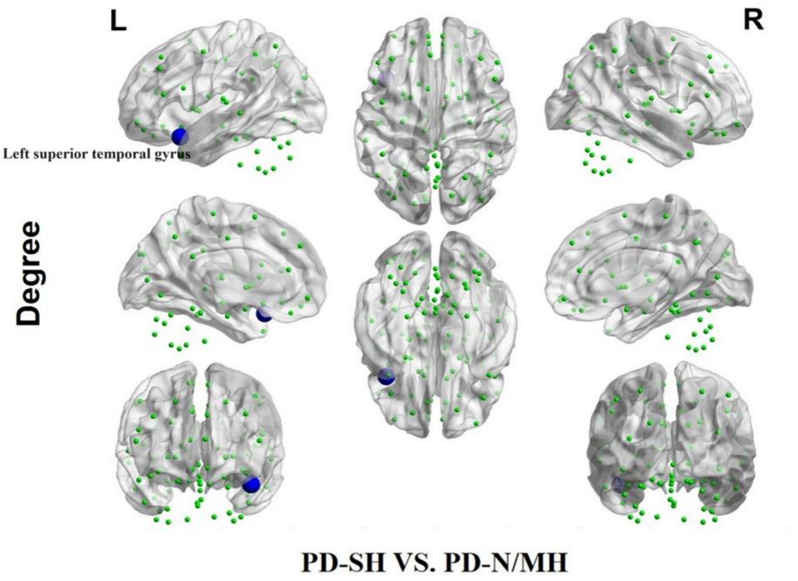

Supplementary Figure 5. The difference in nodal degree between the PD-SH and PD-N/MH group. The blue spheres represent the brain nodes with increased nodal degree in PD-N/MH compared to PD-SH group. R: right; L: left.
